# Incentive Policy Options for Product Remanufacturing: Subsidizing Donations or Resales?

**DOI:** 10.3390/ijerph14121496

**Published:** 2017-12-01

**Authors:** Xiaodong Zhu, Zhe Wang, Yue Wang, Bangyi Li

**Affiliations:** 1 School of Management Engineering, Nanjing University of Information Science Technology, Nanjing 210044, China; zxd@nuist.edu.cn; 2 College of Economics and Management, Nanjing University of Aeronautics and Astronautics, Nanjing 210016, China; wangyue0928@hotmail.com (Y.W.); libangyi@nuaa.edu.cn (B.L.); 3 Odette School of Business, University of Windsor, Windsor, ON N9B 3P4, Canada

**Keywords:** remanufacturing, government subsidy, donation, sales

## Abstract

Remanufactured products offer better environmental benefits, and governments encourage manufacturers to remanufacture through various subsidy policies. This practice has shown that, in addition to product sales, remanufactured product can also achieve its value through social donation. Based on the remanufactured product value realization approaches, governments provide two kinds of incentive policies, which are remanufactured product sales subsidies and remanufactured product donation subsidies. This paper constructs a two-stage Stackelberg game model including a government and a manufacturer under two different policies, which can be solved by backward induction. By comparing the optimal decision of the two policies, our results show that, compared with the remanufacturing sales subsidy, donation subsidy weakens the cannibalization of remanufactured products for new products and increases the quantity of new products. It reduces the sales quantity of remanufactured products, but increases their total quantity. Under certain conditions of low subsidy, the manufacturer adopting sales subsidy provides better economic and environmental benefits. Under certain conditions of high subsidy, the manufacturer adopting donation subsidy offers better economic and environmental benefits. When untreated product environmental impact is large enough, donation subsidy policy has a better social welfare. Otherwise, the choice of social welfare of these two different policies depends on the social impact of remanufactured product donated.

## 1. Introduction

Thanks to technological progress and economic development, products are being upgraded at an accelerated pace. Therefore, the issue of disposing large quantities of end-of-life (EOL) products has become a great concern to our society. Product remanufacturing enables a full utilization of the residual value of EOL products, reduces the demand for energy and resources, and produces favorable economic and environmental benefits [1]. To encourage and support product remanufacturing, governments are willing to subsidize the remanufacturing activities of enterprises. Today, the value of remanufactured products is realized mainly through resales, but some anecdotal data demonstrate that the value can also be realized through product donations. For example, with the approval of the National Development and Reform Commission (NDRC), the Beijing-Xizang Trip event was held in September 2013. During the event, several enterprises (including China National Heavy Duty Truck Group Co., Ltd., Jinan, China and China Shandong Forever Co., Ltd., Zoucheng, China) donated their remanufactured products to the Xizang Autonomous Region. The event was intended to publicize the achievements of the remanufacturing industry and to increase its social acceptance (http://hzs.ndrc.gov.cn/newjsjyxsh/201310/t20131010_561716.html). Due to the effect of “loss aversion”, consumers have a low degree of acceptance towards remanufactured products that are resold in the market, but remanufactured products are particularly suitable for social donations thanks to their characteristics of being of low cost and high quality. Product donations are an important channel through which enterprises can fulfill their social responsibilities [2]. Social donations serve to boost corporate reputation, relieve government pressure and improve social welfare [3,4]. Therefore, governments across the world are supportive of social donations by enterprises. In accordance with the *Tax Law* of China, enterprises can enjoy a tax deduction from their product donations, which represents a sort of indirect government subsidy. This paper presents an incentive policy of subsidizing donations of remanufactured products directly by governments, and also compares it with the prevailing incentive policy of subsidizing the resale of remanufactured products by governments. Furthermore, this paper provides suggestions for government decision-making from the economic, environmental, and social perspectives. This paper mainly addresses the following questions. First, under the two incentive policies, where is the effectiveness boundary for the implementation of original equipment manufacturer (OEM)-based remanufacturing? Second, how do the two subsidy policies influence OEM’s output and pricing decisions? Third, under the two subsidy policies, what are the economic and environmental benefits of OEM-based remanufacturing and which subsidy policy is preferable? Finally, under the two incentive policies, what is the optimal government subsidy level and which incentive policy will bring about higher social welfare?

In order to solve the above questions, this paper constructs a Stackelberg game model with the government being a leader and the manufacturer being a follower, and proposes two kinds of remanufacturing incentive policies including subsidy sales of remanufactured products and subsidy donation of remanufactured products. Under these two kinds of incentive policies, manufacturers establish a mixed production line producing both new and remanufactured products. By solving the equilibrium solution for manufacturers under two incentive policies, the effectiveness boundary of government subsidy is found. From the economic and environmental point of view, we compare the effects of the two subsidy policies on manufacturers, and determine the scope of government subsidies which achieves a “win-win” situation for manufacturer economy and environmental benefits. From the perspective of social welfare, we also give the optimal subsidy level for governments under the two policies, compare the effects of different subsidy policies on the social welfare and attain some meaningful management insights.

Past studies associated with the four questions listed above mainly focus on operation management of remanufacturing, social responsibilities of enterprises and government subsidies. Much of the existing literature studied OEM-based remanufacturing analyzed from the perspective of operation management. These studies cover the issues such as product pricing [5], product quality [6], power construct [7] and channel selection [8]. Subramanian [9] argues that corporate profitability may be adversely affected if remanufacturing is neglected by an OEM’s decisions on common components. Accordingly, Subramanian analyzes how remanufacturing affects the OEM’s decisions on common components, and how the OEM’s profitability is significantly improved if remanufacturing is considered. Yenipazarli [10] proposes a production decision-making model for manufacturer with production and remanufacturing capabilities. He finds that remanufactured products encroach on the markets of new products, while remanufacturing under rational government regulations serves to attain a triple-win situation amongst economic, environmental, and social benefits. According to existing research on double-product-line competitions, the value of remanufactured products is realized only through resales. This paper argues that the value of remanufactured products can be realized through both donations and resales. Many enterprises are also carrying out an increasing level of social responsibility initiatives while generating profits from their production and operation activities. Charitable donations are an important channel through which enterprises can fulfill their social responsibilities. Bekkers [11] describes the driving factors of charitable donations, and Jia [12] finds that charitable donations bestows enterprise with increased government trust and competitive advantage. The above findings are all based on empirical studies. Using a mathematical optimization model, Arya [13] studies the donations and resales of new products by enterprises. Considering the characteristics of remanufactured products, this paper considers remanufactured products in the context of product donations. Governments play an important role in promoting the development of product remanufacturing, and numerous studies have examined government subsidies for resales of remanufactured products. Shu [14] studies the optimal pricing and production decisions of manufacturers under the subsidy or tax rebate policy for product remanufacturing. Specifically, both subsidies and tax rebate are considered favorable by manufacturers, and serve to promote further development of the remanufacturing industry. Xiao [15] studies three subsidy schemes, including subsidy for remanufacturers, subsidy for consumers, and shared subsidy for remanufacturers and consumers. Xiao argues that although subsidies for remanufacturers or consumers can always stimulate remanufacturing activities, subsidies for remanufacturers are the best option. For donations of remanufactured products, governments usually provide matching subsidies (direct subsidies) or rebate subsidies (indirect subsidies) [16]. Eckel [17] finds that matching subsidies or rebate subsidies serve the same function, while rebate subsidies contribute more significantly than matching subsidies. Arya [13] studies government subsidies for donations of new products, demonstrating that direct subsidy can generate the same policy effect as tax reduction. To date, there has been no research concerning government subsidies for donations of remanufactured products. This paper takes government subsidies as a reference point, with the exception that government subsidies are provided to remanufactured products rather than to new products.

In summary, there has been intensive research conducted in literature in the above fields, which lays a good basis for this study. This paper makes the following major contributions to the field, which distinguish it from previous studies: (1) this paper explores new ways for value realization of remanufactured products, that is, the value of remanufactured products can be realized through both donations and resales; (2) this paper designs a new incentive policy for remanufacturing which combines government subsidy with the donations of remanufactured products; (3) this paper compares two distinct incentive policies for remanufacturing from the perspective of production decision, profit, environment and social welfare.

In short, the innovation of this paper is that we consider the “high quality, low cost” characteristics of remanufactured products, extend the way the value of remanufactured products can be realized, propose a new remanufacturing incentive policy which governments subsidize the donations for remanufactured products. Using government subsidies sales of remanufactured products as a benchmark, a more comprehensive comparative analysis of the two kinds of incentive policies is carried out from the perspective of production decision, economic benefits, environmental benefits and social benefits, whereby providing some suggestions to formulate and implement remanufacturing subsidy policy.

The rest of this article is organized as follows: the second section discusses the research questions, research hypotheses and model symbols. The third section builds and solves mathematical models under two policies. The fourth section compares the impact of two different policies from the production decision-making, economic and environmental benefits. The fifth section studies the impact of two subsidy policies from the perspective of social welfare. The sixth section gives some more intuitive numerical examples. At last, we summarize the research results and highlight the management implications from this study.

## 2. Model Depiction and Assumptions

This section mainly describes the hypotheses and notations associated with consumer behaviors, cost structure, government incentives, environmental impact of products, and decision-making sequences used in the rest of the paper.

### 2.1. Consumer Behaviors

Consumers’ demand for product is heterogeneous, and their willingness to pay (ν) is uniformly distributed in the range of [0, 1]. Each consumer purchases at most one unit of the product within a given period, and the market scale is normalized as 1. Due to the effect of loss aversion towards remanufactured products, consumers usually have a lower perception towards remanufactured products than new products. Assume that the willingness to pay is ν for new products and αν for remanufactured products, and αÎ(0,1) indicates the discount of consumers’ perception towards remanufactured products. The price and output of new products are denoted as $p_{n}$ and $q_{n}$, respectively, and the price and output of remanufactured products are denoted as $p_{r}$ and $q_{r}$, respectively. If both new and remanufactured products are available in the market, then the utility of consuming new products is $U_{n}=\nu -p_{n}$ and the utility of consuming remanufactured products is $U_{r}=\alpha \nu -p_{r}$. When $U_{n}>U_{r}$ and $U_{n}>0$, consumers choose to purchase new products, while when $U_{r}>U_{n}$ and $U_{r}>0$, consumers choose to purchase remanufactured products. To ensure remanufactured products are available for sale in the market, a low-price strategy is taken for remanufactured products, namely, $p_{r}<\alpha p_{n}$. Based on the consumer utility function, it can be inferred that the inverse demand functions for new and remanufactured products are $p_{n}=1-q_{n}-\alpha q_{r}$ and $p_{r}=\alpha \left(1-q_{n}-q_{r}\right)$, respectively.

### 2.2. Cost Structure

c indicates the unit cost for new products, and the variable production cost ($c_{r}$) for remanufactured products is denoted by a nonlinear function ($ky^{2}$). The variable production cost for remanufactured products is a convex increasing function with respect to the remanufactured product quantity (y). This shows that more efforts are required to recycle and dispose EOL products [10]. It is assumed that sufficient EOL products are available for recycling and remanufacturing in the market. When manufacturers decide to recycle EOL products for remanufacturing, they need to pay a fixed cost (f) to recycle and dispose of them. After the remanufactured product donation mechanism is carried out, the donation and sale quantity of remanufactured products are denoted as d and $q_{r}$, respectively. Then, the total variable cost of the remanufactured products to be sold and donated can be quantified as $k{\left(q_{r}+d\right)}^{2}$.

### 2.3. Government Incentives

When governments subsidize the resale of remanufactured products, the subsidy for per unit of remanufactured product is denoted as s. When governments subsidize the donation of remanufactured products, the subsidy for per unit of remanufactured product is also denoted as s. The donation of remanufactured products serves to boost corporate reputations, and generates brand value added (b) [13]. Therefore, the benefit of per unit of remanufactured product to be donated can be calculated as s+b. To protect consumer benefits, it is assumed that remanufactured products are always available for sale in the market. Then, s<αc−b. To better analyze the impact of government subsidies for donation of remanufactured products, we also assume that an OEM will not donate their products unless subsidized by governments. Then, we have $b\leq \frac{\alpha ck}{\alpha -\alpha^{2}+k}$.

### 2.4. Environment Impact of Products

The environmental impact of products can be measured by a variety of indices including carbon emissions, hazardous substance content and energy consumption. Today, the vast majority of recycling methods are intended to recycle as many EOL products as possible to reduce landfill and the incineration of EOL products. Therefore, the quantity of non-recycled EOL products can be used to evaluate their environmental impact [18,19]. The smaller the quantity of non-recycled EOL products, the less their environmental impact will be. ξ is denoted as the unit environmental impact of non-recycled products. We assume that the environmental impact is a linearly increasing function with respect to the output of products [10]. Thus, the environmental impact of non-recycled products under two cases is $E^{R}=\xi (q_{n}-q_{r})$ and $E^{D}=\xi (q_{n}-q_{r}-d)$, respectively. To simplify the calculation process, we set ξ=1 [10].

### 2.5. Decision-Making Sequence

This paper builds a two-stage Stackelberg model, in which the regulator seeks to maximize welfare while the manufacturer seeks to maximize profit. The Stackelberg leadership model is a strategic game in Economics in which the leader firm moves first and then the follower firms move sequentially. The German economist Heinrich Freiherr von Stackelberg first proposed this model in 1934 which since then has been applied to study decision-making in various fields of business [8,11,13]. In our model, the regulator is a leader while the manufacturer is a follower. The decision-making sequence is presented as follows: (1) the regulator determines the optimal subsidy level for the two subsidy policies; and (2) the manufacturer determines the quantity of the new and the remanufactured products to be sold or donated taking into consideration the subsidy policies. The model is solved through backward induction: (1) the manufacturer calculates the optimal output levels of new and remanufactured products, respectively; and (2) the regulator calculates the optimal subsidy level under the constraint of welfare maximization according to the OEM’s optimal decisions. The symbols used in this paper are summarized in Table 1.

## 3. The Model

This section presents a decision-making model for OEM’s production, specific to two subsidy schemes: government subsidies for resales of remanufactured products (Policy R), and government subsidies for the donations of remanufactured products (Policy D). The production decision-making model is used to calculate the OEM’s optimal pricing strategy and the quantity of manufactured/remanufactured products under the two subsidy policies.

### 3.1. Policy R: Government Subsidies for the Resales of Remanufactured Products

To encourage OEMs to sell remanufactured products, governments may subsidize the resale of their remanufactured products. Assume that the subsidy level is s. The subsidy amount not only offsets the remanufacturing costs of the OEMs, but also reduces the sale prices of the remanufactured products. It is assumed that market demand is not uncertain, so OEMs can produce the quantity of remanufactured products as demanded by the market. Therefore, governments are subsidizing the production of remanufactured products. Under this subsidy scheme, OEMs’ profit maximization function and environmental objective function are expressed as follows:

(1) $$\underset{\left\{q_{n},q_{r}\right\}}{\max}\Pi^{R}=\left(p_{n}-c\right)q_{n}+\left(p_{r}+s\right)q_{r}-kq_{r}{}^{2}-f$$

(2) $$E^{R}=q_{n}-q_{r}$$

**Proposition** **1.** *Under Policy R, an OEM’s optimal output and prices of new and remanufactured products can be expressed as* $q_{n}^{R*}=\frac{1}{2}\left(1-\frac{c\left(\alpha +k\right)+\alpha s}{\alpha -\alpha^{2}+k}\right)$, $q_{r}^{R*}=\frac{\alpha c+s}{2\left(\alpha -\alpha^{2}+k\right)}$, $p_{n}^{R*}=\frac{1+c}{2}$ *and* $p_{r}^{R*}=\frac{\alpha }{2}+\frac{\alpha \left(ck-\left(1-\alpha \right)s\right)}{2\left(\alpha -\alpha^{2}+k\right)}$, *respectively.*

**Proof.**  There exist optimal solutions to the functions because the Hessian matrix is negative-definite. We can solve the following equations simultaneously, $\frac{\partial \Pi^{R}}{\partial q_{r}}=\alpha -2\left(\alpha +k\right)q_{r}-2\alpha q_{n}+s=0$ and $\frac{\partial \Pi^{R}}{\partial q_{n}}=1-c-2q_{n}-2\alpha q_{r}$. ☐

Considering $q_{n}>0$, the government subsidy amount is $0<s<\frac{\left(1-\alpha -c\right)+\left(1-c\right)k}{\alpha }$. This shows that the equilibrium solutions are economically feasible, and the OEM’s quantity and pricing decisions depend on the parameters k, c and s. As indicated in Table 2, the rise in government subsidies decreases the output levels of new products, but does not affect the prices of new products. However, it increases the output levels of remanufactured products and decrease the prices of remanufactured products. The rise in the costs of new products increases the prices of both new and remanufactured products.

Substituting $q_{n}^{R*}$ and $q_{r}^{R*}$ in Equations (1) and (2), the OEM’s optimal profit is $\Pi^{R*}=\frac{\left(\alpha -k\right){\left(1-c\right)}^{2}+\left(\alpha +s\right)\left(\alpha \left(2c-1\right)+s\right)}{4\left(\alpha -\alpha^{2}+k\right)}-f$, and the optimal environmental impact is $E^{R*}=\frac{1}{2}\left(1-\frac{2\alpha c+ck+\alpha s+s}{\alpha -\alpha^{2}+k}\right)$.

### 3.2. Policy D: Government Subsidies for Donations of Remanufactured Products

In addition to resale, the value of remanufactured products can also be realized through social donations. To encourage an OEM to donate their remanufactured products, governments provide a subsidy (s) for each unit of remanufactured product donated. Under this subsidy scheme, the OEM’s profit maximization function and environmental objective function are expressed as follows:

(3) $$\underset{\left\{q_{n},q_{r},d\right\}}{\max}\Pi^{D}=\left(p_{n}-c\right)q_{n}+p_{r}q_{r}-k{\left(q_{r}+d\right)}^{2}+\left(s+b\right)d-f$$

(4) $$E^{D}=q_{n}-\left(q_{r}+d\right)$$

**Proposition** **2.** *Under Policy D, the OEM’s optimal output and price of new and remanufactured products can be expressed as* $q_{n}^{D*}=\frac{1-\alpha -c+b+s}{2-2\alpha }$*,* $q_{r}^{D*}=\frac{\alpha c-(b+s)}{2\left(1-\alpha \right)\alpha }$*,* $d^{D*}=\frac{1}{2}\left(\frac{b-c+s}{1-\alpha }+\frac{b+s}{k}+\frac{b+s}{\alpha }\right)$*,* $p_{n}^{D*}=\frac{1+c}{2}$ *and* $p_{r}^{D*}=\frac{\alpha +b+s}{2}$*, respectively.*

**Proof.**  There exist optimal solutions to the functions because the Hessian matrix is negative-definite. We can solve the following equations simultaneously, $\frac{\partial \Pi^{R}}{\partial q_{r}}=\alpha -2dk-2\left(\alpha +k\right)q_{r}-2\alpha q_{n}=0$, $\frac{\partial \Pi^{R}}{\partial q_{n}}=1-c-2q_{n}-2\alpha q_{r}$ and $\frac{\partial \Pi^{R}}{\partial d}=b-2k\left(d+q_{r}\right)+s$. ☐

Considering $q_{r}^{D*}>0$ and $d^{D*}>0$, the government subsidy amount is $\frac{\alpha ck}{\alpha -\alpha^{2}+k}-b<s<\alpha c-b$. This shows that the equilibrium solutions are economically feasible. According to $\frac{\alpha ck}{\alpha -\alpha^{2}+k}-b\geq 0$, the corporate reputation from donations is $b\leq \frac{\alpha ck}{\alpha -\alpha^{2}+k}$, and the OEM’s output and pricing decisions depend on the parameters k, c, s and b. Under Policy *D*, government subsidies and corporate reputations from product donations increase the output of new products, but do not affect the pricing of new products (as indicated in Table 3). Meanwhile, they reduce the resale quantity of remanufactured products, increase the donation quantity of remanufactured products, and increase the resale price of remanufactured products. The increase in the cost of remanufacturing only reduces the donation quantity of remanufactured products. The increase in the cost of new products reduces the quantity of new products and donation quantity of remanufactured products, but increases the sale quantity of manufactured products.

Substituting $q_{n}^{D*}$ and $q_{r}^{D*}$ in Equations (3) and (4), the OEM’s optimal profit is $\Pi^{D*}=\frac{\alpha k\left(1-\alpha +c\left(2\alpha +c-2\right)\right)+{\left(b+s\right)}^{2}\left(\alpha -\alpha^{2}+k\right)-2\alpha ck\left(b+s\right)}{4\left(1-\alpha \right)\alpha k}-f$, and the optimal environmental impact is $E^{D*}=\frac{1}{2}\left(\frac{b+s-c}{1-\alpha }-\frac{b+s}{k}+1\right)$.

## 4. Analysis

This section comparatively analyzes the two subsidy schemes in terms of three aspects: the OEM’s output and pricing decisions, economic benefits, and environmental benefits. We discuss the common subsidy level shared by the two subsidies schemes, and compare the lower limit of government subsidies under the two subsidy schemes. The lower limit ($s^{L}$) of government subsidies depends on the corporate reputation from donations of remanufactured products. Then, we obtain $s^{L}=\max\{\frac{\alpha ck}{\alpha -\alpha^{2}+k}-b,0\}$ and $0<b\leq \frac{\alpha ck}{\alpha -\alpha^{2}+k}$. We also compare the upper limit of government subsidies between the two subsidy schemes. The upper limit of government subsidies is $s^{U}=\min\{\alpha c-b,\frac{\alpha \left(1-\alpha -c\right)-ck+k}{\alpha }\}$. When the costs of new products meet the condition $c>\frac{\alpha \left(1-\alpha +b\right)+k}{\alpha^{2}+\alpha +k}$, we have $\alpha c-b>\frac{\alpha \left(1-\alpha -c\right)-ck+k}{\alpha }$; otherwise, we have $\alpha c-b<\frac{\alpha \left(1-\alpha -c\right)-ck+k}{\alpha }$. When the production costs of new products are high, the opportunity costs of donations of remanufactured products increase. Therefore, higher subsidies are required for remanufactured products to be donated. In sum, the common subsidy level shared by the two subsidy schemes is $s^{L}<s<s^{U}$. We note that when $b<\frac{\alpha ck}{\alpha -\alpha^{2}+k}$, only Policy *R*, rather than Policy *D*, exists in the range of $\left(0,\frac{\alpha ck}{\alpha -\alpha^{2}+k}-b\right)$.

### 4.1. Comparison of OEM’s Equilibrium Solutions

This section compares the output and pricing decisions between Policys *D* and *R*. Let $\Delta \cdot =\Delta \cdot^{D}-\Delta \cdot^{R}$, indicating the difference of parameter ⋅ between Policys *D* and *R*. Here, ⋅ indicates the output and price of different products.

**Proposition** **3.** *Table 4 lists the differences of output and pricing decisions with respect to new and remanufactured products in Policys D and R. “+” indicates *Δ⋅>0, *“−” indicates* Δ⋅<0, *and “0” indicates* Δ⋅=0*.*

According to Proposition 3, government subsidies for donations of remanufactured products increase the output of both new and remanufactured products, but decrease the sale quantity of remanufactured products. This is due to two reasons: (1) donations of remanufactured products encroach on the resale markets of remanufactured products, thus decreasing the sale quantity of remanufactured products; and (2) donations of remanufactured products weaken the encroachment on the output of new products by the resales of remanufactured products, thus increasing the sale quantity of new products. What is noteworthy noting is that the prices of new products remain unchanged despite the increase in the sale quantity of new products. The donation of remanufactured products bring about a premium effect to the resale pricing of remanufactured products, thus reducing consumer benefits to a certain degree.

### 4.2. Comparison of OEM’s Profits and Environment Impacts

To better understand the effect of the two subsidy schemes on OEM’s profits and environmental impacts, and assist in selecting a better subsidy scheme, we use $\Delta \Pi^{D-R}(s)=\Pi^{D*}-\Pi^{R*}$ and $\Delta E^{D-R}(s)=E^{D*}-E^{R*}$, respectively to denote the differences in profits and environmental benefits between the two subsidy schemes.

(1) Comparison of Profits

We obtain the result of $\Delta \Pi^{D-R}(s)=\frac{1}{4}\left(\frac{{\left(b+s-c\right)}^{2}}{1-\alpha }+\frac{{\left(b+s\right)}^{2}}{k}+\frac{{\left(b+s\right)}^{2}}{\alpha }-\frac{c^{2}\left(\alpha +k\right)+2\alpha cs+s^{2}}{\alpha -\alpha^{2}+k}\right)$, and calculate the first and second derivatives of s regarding $\Delta \Pi^{D-R}(s)$. This yields Proposition 4 as follows.

**Proposition** **4.** $\Delta \Pi^{D-R}(s)$ *is a convex function in* s*, and is monotonically decreasing in the range* $\left(s^{L},s_{1}\right)$*, but monotonically increasing in the range* $\left(s_{1},s^{U}\right)$*.* $s_{1}$ *is its minimum value. Regarding* $\Delta \Pi^{D-R}(s)=0$*, there exists a unique root* $s_{2}$*, namely,* $\Delta \Pi^{D-R}(s_{2})=0$*. Specifically,* $s_{1}=\frac{\alpha ck\left(k+2\left(1-\alpha \right)\alpha \right)-b{\left(\alpha -\alpha^{2}+k\right)}^{2}}{\left(1-\alpha \right)\alpha \left(\left(1-\alpha \right)\alpha +k\right)+k^{2}}$*,* $s_{2}=s_{1}+\frac{\left(1-\alpha \right)\alpha k\left(\alpha -\alpha^{2}+k\right)\Theta }{\left(1-\alpha \right)\alpha \left(\left(1-\alpha \right)\alpha +k\right)+k^{2}}$ *and* $\Theta =\sqrt{\frac{3\alpha^{2}c^{2}k+b^{2}\left(\alpha -\alpha^{2}+k\right)-2\alpha bc\left(\alpha -\alpha^{2}+2k\right)}{\left(1-\alpha \right)\alpha k\left(\alpha -\alpha^{2}+k\right)}}$*.*

**Proof.**  The first derivative is $\frac{\partial \Delta \Pi^{D-R}(s)}{\partial s}=\frac{2\left(b-c+s\right)}{1-\alpha }+\frac{2\left(b+s\right)}{k}+\frac{2\left(b+s\right)}{\alpha }-\frac{2\alpha c+2s}{\alpha -\alpha^{2}+k}=0$, and thus we obtain the extreme point $s_{1}$ ($s_{1}\in (s^{L},s^{U})$). We calculate the second derivative $\frac{\partial^{2}\Delta \Pi^{D-R}(s)}{\partial s^{2}}=2\left(\frac{k}{\left(\alpha -1\right)\alpha \left(\left(\alpha -1\right)\alpha -k\right)}+\frac{1}{k}\right)>0$; the extreme point $s_{1}$ is also the max-min point. Let $\Delta \Pi^{D-R}(s)=0$, and thus calculate $s_{2}$ ($s_{1}<s_{2}\in (s^{L},s^{U})$). ☐

According to Proposition 4, $\Delta \Pi^{D-R}(s)$ is monotonically decreasing in the range $\left(s^{L},s_{1}\right)$. Therefore, compared to Policy *D*, a low subsidy level has a more significant influence on profit under Policy *R*, where profit under Policy *R* is higher than under Policy *D*. $\Delta \Pi^{D-R}(s)$ is monotonically increasing in the range $\left(s_{1},s^{U}\right)$. Therefore, when the subsidy level reaches $s_{1}$, the influence of government subsidies on profit under Policy *D* is increasing. The difference in profits between Policys *D* and *R* diminishes as the subsidy level rises. When $s>s_{2}$, profit under Policy *D* is higher than under Policy *R*.

(2) Comparison of Environmental Impacts

We obtain the result of $\Delta E^{D-R}(s)=\frac{1}{2}\left(\frac{b-c+s}{1-\alpha }-\frac{b+s}{k}+\frac{2\alpha c+ck+\alpha s+s}{\alpha -\alpha^{2}+k}\right)$, and calculate the first and second derivatives of s regarding $\Delta E^{D-R}(s)$. This yields Proposition 5.

**Proposition** **5.** $\Delta E^{D-R}(s)$ *is monotonically decreasing in the range* $\left(s^{L},s^{U}\right)$*.* *There exists a unique root* $s_{3}$*, which ensures* $\Delta E^{D-R}(s_{3})=0$*. Thereby,* $\Delta E^{D-R}(s)>0$ *in* $s\in (s^{L},s_{3})$ *and* $\Delta E^{D-R}(s)<0$ *in* $s\in (s_{3},s^{U})$*. Here,* $s_{3}=\frac{\left(1-\alpha -k\right)\left(\alpha ck-b\left(\alpha -\alpha^{2}+k\right)\right)}{\left(1-\alpha \right)\alpha \left(1-\alpha -2k\right)-k^{2}}$*.*

**Proof.**  The first derivative of $\Delta E^{D-R}(s)$ is $\frac{\partial \Delta E^{D-R}(s)}{\partial s}=\frac{\left(1-\alpha \right)\alpha \left(1-\alpha -2k\right)-k^{2}}{2\left(\alpha -1\right)k\left(\alpha -\alpha^{2}+k\right)}<0$. According to $\Delta E^{D-R}(s)=0$, we can calculate the root $s_{3}\in (s^{L},s^{U})$. ☐

According to Proposition 5, $\Delta E^{D-R}(s)$ monotonically decreases as government subsidies increase, indicating that environmental impact continues to improve. With an increase in government subsidies, the quantity of remanufactured products increases while the quantity of non-recycled products decreases. At a low subsidy range ($s\in (s^{L},s_{3})$), the environmental impact under Policy *R* is superior to that under Policy *D*. At a high subsidy range ($s\in (s_{3},s^{U})$), the environmental impact under Policy *D* is superior to that under Policy *R*. This is due to the fact that donations of remanufactured products increase not only the total output of remanufactured products, but also the output of new products. At a low subsidy range, Policy *D* is inferior to Policy *R* in terms of environmental impact if the increment in remanufactured products arising from the donations of remanufactured products is smaller than the increment in new products. At a high subsidy range, Policy *D* is superior to Policy *R* in terms of environmental impact if the increment in remanufactured products arising from the donations of remanufactured products is larger than the increment in new products.

**Proposition** **6.** *When the subsidy level is in the range* $s\in (s_{2},s^{U})$*, Policy D is superior to Policy R in terms of both environmental and economic benefits, and thus there exists a win-win scenario. When the subsidy range is in the range* $s\in (s^{L},s_{3})$*, Policy R is superior to Policy D in terms of both environmental and economic benefits, and thus there exists a win-win scenario. When the subsidy level is in the range* $s\in (s_{3},s_{2})$*, Policy R is superior to Policy D in terms of economic benefits, while Policy D is superior to Policy R in terms of environmental benefits.*

**Proof.**  Because $s_{2}=s_{1}+\frac{\left(1-\alpha \right)\alpha k\left(\alpha -\alpha^{2}+k\right)\Theta }{\left(1-\alpha \right)\alpha \left(\left(1-\alpha \right)\alpha +k\right)+k^{2}}$, $s_{2}-s_{3}$ can be converted into $s_{1}-s_{3}$. According to the calculation, $s_{1}>s_{3}$, thus proving that $s_{2}>s_{3}$. ☐

Proposition 6 provides the basis for selecting one of the two subsidy policies. When remanufactured products are subsidized by the government, the subsidy level should not fall within the range $s\in (s_{3},s_{2})$. In such a range, neither of the two subsidy policies generate a win-win scenario in terms of economic and environmental benefits. When there is insufficient budget for government subsidies, namely, $s\in (s^{L},s_{3})$, it is recommended that government subsidies be provided to the resale of remanufactured products. The intent is to attain a win-win scenario in terms of economic and environmental benefits to the low satisfaction result of OEMs and governments. When there is sufficient budget for government subsidies, namely, $s\in (s_{2},s^{U})$, it is recommended that government subsidies be provided to the donations of remanufactured products. The intent is to attain a win-win scenario in terms of economic and environmental benefits to the high satisfaction result of OEMs and governments.

## 5. Social Welfare

To attain a better balance among economic, environmental, and social benefits, governments as the regulator determine the optimal subsidy level for the purpose of maximizing social welfare. Using the social welfare model (He et al. [20,21]) as a reference, this section analyzes the impact of government subsidies on government interests, OEM profits, consumer surplus and environmental benefits, as well as the social impact of product donations.

### 5.1. Social Welfare of Policy R

$SW^{R}(s)=G^{R*}+\Pi^{R*}+CS^{R*}+\omega (E^{R*})$ indicates the social welfare maximization function under Policy *R*. In this equation, $G^{R}=-sq_{r}^{R*}$ indicates the government subsidies for resale of remanufactured products, $\Pi^{R*}$ indicates the OEM profits, $CS^{R*}=\frac{{\left(q_{n}^{R*}+\alpha q_{r}^{R*}\right)}^{2}+\alpha \left(1-\alpha \right)q_{r}^{R{*}^{2}}}{2}$ indicates the consumer surplus, and $\omega (E^{R*})=e(q_{n}-q_{r})$ indicates the total environmental cost of non-recycled products. e indicates the unit environmental cost of non-recycled products. The following result is obtained:

(5) $$SW^{R}(s)=q_{n}^{R*}\left(1-c-e-\alpha q_{r}^{R*}\right)+\left(\alpha +e\right)q_{r}^{R*}-\frac{1}{2}\left(\alpha +2k\right)q_{r}^{R{*}^{2}}-\frac{q_{n}^{R{*}^{2}}}{2}$$

**Proposition** **7.** *Under Policy R, there exists a unique optimal subsidy level* $s=s_{4}$ *where,* $s_{4}=\frac{2e\left(\alpha -\alpha^{3}+\alpha k+k\right)+\left(1-\alpha \right)\alpha^{2}c}{\alpha -\alpha^{2}+2k}$*.*

**Proof.**  It can be shown that $\frac{\partial^{2}SW^{R}(s)}{\partial s^{2}}<0$ since $SW^{R}(s)$ is a strictly concave function, and there exists a unique optimal value in the range $0<s<\frac{\left(1-\alpha -c\right)+\left(1-c\right)k}{\alpha }$. ☐

According to Proposition 7, social welfare under Policy *R* is a strictly concave function with respect to government subsidies, and there exists a unique optimal subsidy level. The government subsidy level increases as the costs of new products, costs of remanufacturing, and external impact of products all increase.

**Corollary.** **1.** Regardless of the degree of external environmental impact of products, governments must subsidize the remanufactured products under Policy R.

**Proof.**  Under the conditions e>0 and 0<α<1, it can be shown that $s_{4}=\frac{2e\left(\alpha -\alpha^{3}+\alpha k+k\right)+\left(1-\alpha \right)\alpha^{2}c}{\alpha -\alpha^{2}+2k}>0$. ☐

Corollary 1 shows that government subsidies for resales of remanufactured products are less affected by the externality of product environments. Regardless of the degree of environmental impact of products, governments should subsidize the remanufacturing behaviors of the OEM to encourage remanufacturing and enhance environmental benefits.

### 5.2. Social Welfare of Policy D

$SW^{D}(s)=G^{D*}+\Pi^{D*}+CS^{D*}+\omega (E^{D*})+\Phi d^{D{*}^{2}}$ indicates the social welfare maximization function regarding government subsidies for donations of remanufactured products. In the function, $G^{D}=-sd_{}^{D*}$ indicates the government subsidy level for donations of remanufactured products, $\Pi^{D*}$ indicates the OEM’s profits, $\Pi^{D*}$ indicates the consumer surplus, $\omega (E^{D*})=e\left(q_{n}-(q_{r}+d)\right)$ indicates the environmental impact of non-recycled products, $\Phi d^{D{*}^{2}}$ indicates the social impact from the donations of remanufactured products, and Φ>0 indicates the social impact coefficient of the donations of remanufactured products.

(6) $$SW^{D}(s)=q_{n}^{D*}\left(1-c-e-\alpha q_{r}^{D*}\right)+\left(\alpha +e\right)q_{r}^{D*}-\frac{q_{n}^{D{*}^{2}}}{2}-\frac{\alpha q_{r}^{D{*}^{2}}}{2}+\Phi d^{D{*}^{2}}-k{\left(d_{}^{D*}+q_{r}^{D*}\right)}^{2}+d_{}^{D*}\left(b+e\right)$$

**Proposition** **8.** *When* $\Phi <\Phi_{1}$*, there exists a unique optimal subsidy level* $s=s_{5}$ *under Policy D. When* $\Phi >\Phi_{1}$*, there exists no optimal subsidy level under Policy D. Specifically,* $s_{5}=\frac{\left(1-\alpha \right)\alpha k\left(bk+\alpha \left(2e\left(1-\alpha -k\right)-ck\right)\right)+2\Phi \left(\alpha -\alpha^{2}+k\right)\left(\alpha ck-b\left(\alpha -\alpha^{2}+k\right)\right)}{2\Phi {\left(\alpha -\alpha^{2}+k\right)}^{2}+\left(\alpha -1\right)\alpha k\left(k-2\left(\alpha -1\right)\alpha \right)}$ *and* $\Phi_{1}=\frac{\left(1-\alpha \right)\alpha k\left(2\alpha -2\alpha^{2}+k\right)}{2{\left(\alpha -\alpha^{2}+k\right)}^{2}}$*.*

**Proof.**  When $\Phi <\Phi_{1}$, we have $\frac{\partial^{2}SW^{D}(s)}{\partial s^{2}}<0$. When this holds, $SW^{D}(s)$ is a strictly concave function, and there exists a unique maximum value $s_{5}$. When $\Phi >\Phi_{1}$, we have $\frac{\partial^{2}SW^{D}(s)}{\partial s^{2}}>0$. When this holds, $SW^{D}(s)$ is a strictly convex function, and the known effective subsidy level is in an open interval of $\left(\frac{\alpha ck}{\alpha -\alpha^{2}+k}-b,\alpha c-b\right)$. Therefore, the optimal value is not available at the endpoints. ☐

According to Proposition 8, the social impact coefficient (Φ) of donations of remanufactured products determines whether there exists an optimal subsidy level under Policy *D*. When the value of Φ is small, there exists a unique optimal subsidy level. It is worth noting that the optimal subsidy level decreases as the costs of new products increase, but increases as the external impact of products increases. It is uncertain and is affected by the costs of remanufacturing.

**Corollary** **2.** *When* $\Phi <\Phi_{1}$*, government subsidies are necessary only when the environmental externality of products meets the condition $e>\frac{\alpha ck\left(2k+3\left(1-\alpha \right)\alpha \right)-2b{\left(\alpha -\alpha^{2}+k\right)}^{2}}{2\alpha \left(1-\alpha -k\right)\left(\left(1-\alpha \right)\alpha +k\right)}$ under Policy D.*

**Proof.**  Under the conditions $\Phi <\Phi_{1}$ and $s_{5}-s^{L}>0$, it can be shown that $e>\frac{\alpha ck\left(2k+3\left(1-\alpha \right)\alpha \right)-2b{\left(\alpha -\alpha^{2}+k\right)}^{2}}{2\alpha \left(1-\alpha -k\right)\left(\left(1-\alpha \right)\alpha +k\right)}$. ☐

Compared with Corollary 1, government subsidies for donations of remanufactured products are less affected by the external environment impact of non-recycled products. In other words, government subsidies are necessary only when the external environmental impact of non-recycled products reaches a certain level. Otherwise, it is not necessary for governments to intervene in the production and remanufacturing actions of the OEM.

**Proposition** **9.** *When* $e>e_{1}$*, we have* $\Phi_{2}<0$ *and* $SW^{D}(s)>SW^{R}(s)$*. When* $e<e_{1}$*, we have* $\Phi >\Phi_{2}$ *and* $SW^{D}(s)>SW^{R}(s)$*. When* $0<\Phi <\Phi_{2}$*, we have* $SW^{D}(s)<SW^{R}(s)$*.*

**Proof.**  Let $\Delta SW=SW^{D}(s)-SW^{R}(s)=0$. Then, we obtain the result $\Phi =\Phi_{2}=\frac{G^{R*}+\Pi^{R*}+CS^{R*}+E^{R*}-(G^{D*}+\Pi^{D*}+CS^{D*}+E^{D*})}{d^{D{*}^{2}}}$. When $\Phi >\Phi_{2}$, we have ΔSW>0; otherwise, ΔSW<0. Let $\Omega =G^{R*}+\Pi^{R*}+CS^{R*}+E^{R*}-(G^{D*}+\Pi^{D*}+CS^{D*}+E^{D*})$, and solve Ω=0. Thus, we obtain the result $e=e_{1}$. It can be shown that when $e>e_{1}$, Ω<0, and Ω>0 otherwise. When $e>e_{1}$, $\Phi_{2}<0$; given $\Phi >0>\Phi_{2}$, ΔSW>0. When $e<e_{1}$, $\Phi_{2}>0$; when $\Phi >\Phi_{2}$, ΔSW>0; when $0<\Phi <\Phi_{2}$, ΔSW<0; where, $e_{1}=\frac{b^{2}{\left(\alpha -\alpha^{2}+k\right)}^{2}\left(3k-2\left(\alpha -1\right)\alpha \right)+2bk{\left(\alpha -\alpha^{2}+k\right)}^{2}\left(s-3\alpha c\right)+2\left(\alpha -1\right)\alpha k^{2}\left(2\alpha cs+s^{2}-2\alpha^{2}c^{2}\right)-k^{3}\left(s-\alpha c\right)\left(3\alpha c+s\right)-4{\left(\alpha -1\right)}^{2}\alpha^{2}ks\left(\alpha c+s\right)+2{\left(\alpha -1\right)}^{3}\alpha^{3}s^{2}}{4\alpha \left(\left(\alpha -1\right)\alpha -k\right)\left(b\left(1-\alpha -k\right)\left(\alpha -\alpha^{2}+k\right)+k^{2}\left(\alpha c-s\right)+\alpha \left(\alpha -1\right)k\left(c+2s\right)+\alpha {\left(\alpha -1\right)}^{2}s\right)}$. ☐

According to Proposition 9, government subsidies for donations of remanufactured products will improve social welfare if the external environmental impact of non-recycled products is significant. Therefore, the more significant the external environmental impact of non-recycled products, the more feasible the government subsidies will be for donations of remanufactured products. If the external environmental impact of non-recycled products is minimal, the social impact from donations of remanufactured products determines which subsidy policy will improve social welfare more. From the perspective of social welfare, government subsidies for donations of remanufactured products are preferable if the social benefits from donations of remanufactured products are significant enough. Otherwise, government subsidies for resales of remanufactured products are preferable.

## 6. Numerical Example

This section analyzes the economic and environmental benefits of product remanufacturing under the two different subsidy policies using numerical examples. The analysis not only corroborates propositions and conclusions set forth above, but also offers management insights to us. The parameter values in the production decision-making model used in the analysis are as follows: α=0.6, k=0.01, c=0.1, c=0.26 and b=0.002. Considering that the government subsidy level differs between the two subsidy schemes, we conduct a numerical analysis under two circumstances: high and low production costs of new products. When c=0.1, the common subsidy range shared by the two subsidy schemes is 0.0004<s<0.058. To facilitate graphical presentation, we select the subsidy range 0.0004<s<0.01. When c=0.26, the common subsidy range shared by the two subsidy schemes is 0.00424<s<0.15233. To facilitate graphical presentation, we select the subsidy range of 0.00424<s<0.03.

**Figure 1:**
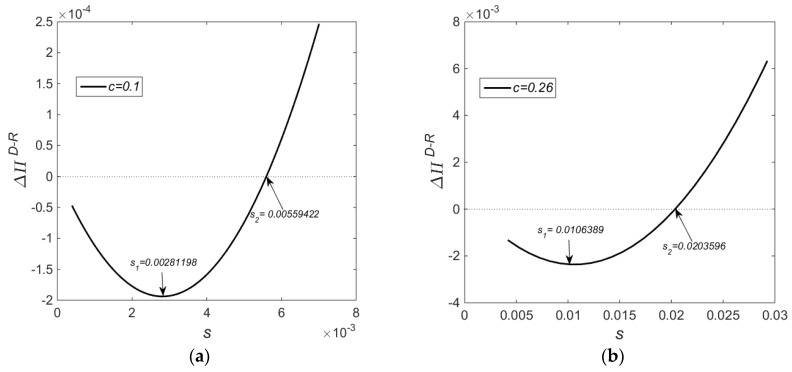
Impact on the Profit Difference from Two Subsidy Models for Different Subsidy Levels; (**a**) c=0.1; (**b**) c=0.26.

Figure 1 shows the impact on the difference in profit from the two subsidy schemes for different subsidy ranges. The profit difference function is a convex function with respect to government subsidies which decreases initially and then increases as government subsidies increase. When c=0.1, there exists a minimum value (s=0.00281198) in terms of the subsidy range, and when c=0.26, there exists a minimum value (s=0.0106389) in terms of the subsidy range. With the rise in production costs of new products, the subsidy range also increases accordingly.

**Figure 2:**
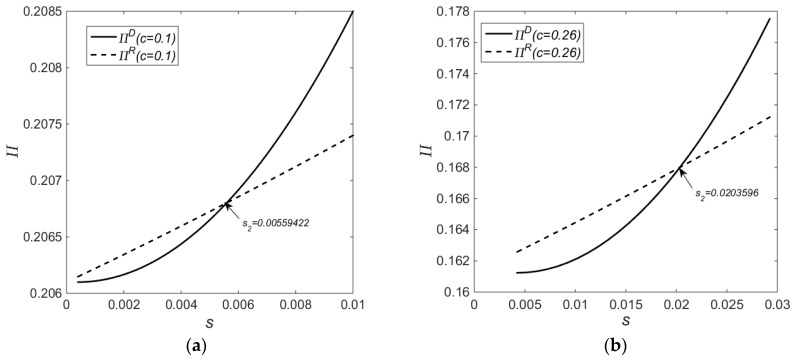
Impact on the OEM’s Profits by Different Subsidy Levels; (**a**) c=0.1; (**b**) c=0.26.

Figure 2 shows the impact of different subsidy levels on economic benefits. According to Figure 2 as well as Figure 1, when the subsidy level is sufficiently low, the subsidy level affects Policy *R* more significantly than Policy *D*, and the profits under Policy *R* grow more rapidly than those under Policy *D*. In this situation, Policy *R* is superior to Policy *D* in terms of economic benefits. When the subsidy level reaches $s_{1}$, the subsidy level affects Policy *D* more significantly than Policy *R* while the profits under Policy *R* are still higher than those under Policy *D*. Therefore, the profit difference between the two subsidy schemes is a convex function with respect to government subsidies. When the subsidy level reaches $s_{2}$, the profits under Policy *D* grow more rapidly than those under Policy R, and Policy *D* begins to exceed Policy *R* in terms of economic benefits.

**Figure 3:**
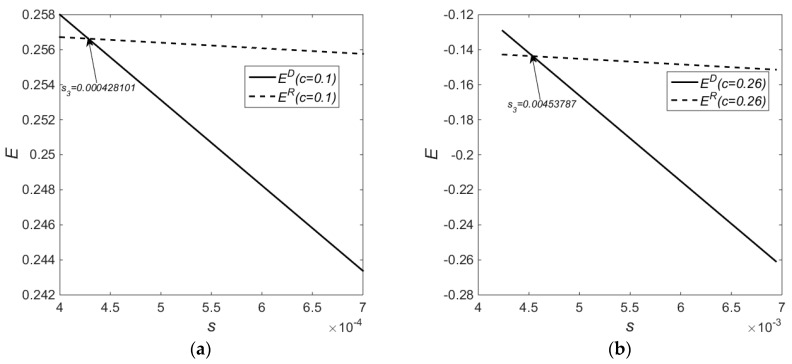
Impact on Environmental Benefits by Different Subsidy Levels; (**a**) c=0.1; (**b**) c=0.26.

Figure 3 shows the impact on environmental benefits by different subsidy levels. When the subsidy level is sufficiently low, the environmental benefits under Policy *D* are inferior to those under Policy *R*. When the subsidy level reaches $s_{3}$, the environmental benefits under Policy *D* are superior to those under Policy *R*. It is worth noting that when the costs of new products are very high (c=0.26) and the costs of remanufacturing is very low (c=0.01), the quantity of new products decreases, the quantity of remanufactured products increases, and remanufacturing gains benefits more significantly. This paper assumes that the quantity of EOL products is sufficient in the market, so the quantity of remanufactured products may exceed the quantity of new products in the numerical analysis of the example. As a result, both subsidy schemes generate enhanced environmental benefits after remanufacturing is introduced.

Concluding from Figure 1, Figure 2 and Figure 3 collectively, at a low-cost level and in the subsidy range s∈(0.0004,0.00428101), there exists a win-win scenario of economic and environmental benefits under Policy *R* as compared to Policy *D*. At a high cost level and in the subsidy range s∈(0.00424,0.00453787), there exists a win-win scenario of economic and environmental benefits under Policy *R* as compared to Policy *D*. At a low-cost level and in the subsidy range s∈(0.00559422,0.058), there exists a win-win scenario of economic and environmental benefits under Policy *D* as compared to Policy *R*. Finally, at a high cost level and in the subsidy range s∈(0.0203596,0.15233), there exists a win-win scenario of economic and environmental benefits under Policy *D* as compared to Policy *R*.

## 7. Conclusions

In previous studies, the value of remanufactured products was realized only through resales. Real life practice shows that the value of remanufactured products can also be realized through social donations. To encourage and support the remanufacturing of EOL products, this paper considers two incentive policies (government subsidies for resales of remanufactured products and for donations of remanufactured products). We build a two-stage game model involving both government and OEM which is specific to the two incentive policies, and calculate the equilibrium solutions to analyze the impact of the two subsidy schemes on the OEM’s production decisions, and environmental and economic benefits. From the perspectives of economic, environmental and social benefits, we examine how to choose incentive policies and determine the optimal subsidy range.

The research findings of this study are summarized as follows. First, under the two incentive policies, the effectiveness boundary of government subsidies is different from each other, while there exists a common subsidy range $s^{L}<s<s^{U}$. Second, when the government subsidizes the resales of remanufactured products, the subsidies reduce the sale quantity of new products, and increases the sale quantity of remanufactured products, thus aggravating the market encroachment of remanufactured products on new products. When the government subsidizes the donations of remanufactured products, the subsidies increase the sale quantity of both new products and the donation quantity of remanufactured products, but reduces the sale quantity of remanufactured products, thus alleviating the market encroachment of remanufactured products on new products. Third, government subsidies for donations of remanufactured products reduce the sale quantity of remanufactured products, but increases the total quantity of new products and remanufactured products. Fourth, at a low level, government subsidies for resales of remanufactured products generate a win-win scenario of economic and environmental benefits within a certain subsidy range. At a high level, government subsidies for donations of remanufactured products generate a win-win scenario of economic and environmental benefits within a certain subsidy range. Fifth, for government subsidies for resales of remanufactured products, there exists a unique optimal subsidy range; governments will always provide subsidies regardless of the degree of external environmental impact of products. When the benefits from the donations of remanufactured products meet the condition $\Phi <\Phi_{1}$, there exists a unique optimal subsidy level regarding the subsidies for donations of remanufactured products. In addition, government subsidies are not necessary unless the external environmental impact of the non-recycled products is significant enough. Sixth, when the external environmental impact of products is significant enough, government subsidies for donations of remanufactured products improve social welfare more than government subsidies for resales of remanufactured products. When the external environmental impact of products is not significant enough, the social benefits (Φ) from the donations of remanufactured products determine which incentive policy will improve social welfare.

A two-stage Stackelberg model is built and solved based on complete information. Problem analysis will become more complex if incompleteness of information is assumed in the model. Meanwhile, this paper only considers the OEM’s single-period decisions. New findings will be obtained if the Stackelberg model considers two-period and multi-period decisions as well as the constraints of remanufacturing. This will be further investigated in our subsequent research.

## Figures and Tables

**Table 1:** Description of Related Symbols.

Symbol	Description
Decision variables	
$q_{n},p_{n}$	Sales quantity and price of new products
$q_{r},p_{r}$	Sales quantity and price of remanufactured products
d	Donation quantity of remanufactured products
Parameter	
c	Production cost of new products
$c_{r}=ky^{2}$	Total variable cost of y units of remanufactured products
f	Fixed capital cost for remanufacturing
α	Consumer perception discount towards remanufactured products
b	Brand reputation
Φ	Social impact coefficient for the donations of remanufactured products
ξ	Unit environmental impact of non-recycled products
E	Environmental impact of non-recycled products
G	Government subsidies
Π	OEM profits
CS	Consumer surplus
e	Unit environmental cost of non-recycled products
ω	Total environmental cost of non-recycled products
SW	Social welfare

**Table 2:** Influence of Various Parameters under Policy *R* on the OEM’s Output and Pricing Decisions.

	Variable	$q_{n}^{R*}$	$p_{n}^{R*}$	$q_{r}^{R*}$	$p_{r}^{R*}$
Parameter					
k↗		↗	×	↘	↗
c↗		↘	↗	↗	↗
s↗		↘	×	↗	↘

The signs ↗, ↘ and × represent the relationships as being monotonic increasing, monotonic decreasing and unrelated with respect to the parameter, respectively.

**Table 3:** Influence of Various Parameters under Policy *D* on the OEM’s Output and Pricing Decisions.

	Variable	$q_{n}^{D*}$	$p_{n}^{D*}$	$q_{r}^{D*}$	$p_{r}^{D*}$	$d^{D*}$
Parameter						
k↗		×	×	×	×	↘
c↗		↘	↗	↗	×	↘
s↗		↗	×	↘	↗	↗
b↗		↗	×	↘	↗	↗

The signs ↗, ↘ and × represent the relationships as being monotonic increasing, monotonic decreasing and unrelated with respect to the parameter, respectively.

**Table 4:** Comparison of OEM’s Equilibrium Solutions Between Policys *D* and *R.*

Δ⋅	$\Delta q_{n}$	$\Delta p_{n}$	$\Delta q_{r}$	$\Delta p_{r}$	Δd	$\Delta (d+q_{r})$
Sign	+	0	−	+	+	+
